# Do pet dogs (*Canis familiaris*) follow ostensive and non-ostensive human gaze to distant space and to objects?

**DOI:** 10.1098/rsos.170349

**Published:** 2017-07-26

**Authors:** Charlotte Duranton, Friederike Range, Zsófia Virányi

**Affiliations:** 1 Laboratoire de Psychologie Cognitive (UMR 7290), Aix-Marseille University and CNRS, Fédération 3C, Marseilles, France; 2 Ferme du Quesnoy, AVA Association, 76220 Cuy-Saint-Fiacre, France; 3 Clever Dog Lab, Comparative Cognition, Messerli Research Institute, University of Veterinary Medicine, Vienna, Medical University of Vienna, University of Vienna, Vienna, Austria; 4 Wolf Science Center, Messerli Research Institute, University of Veterinary Medicine, Vienna, Medical University of Vienna, University of Vienna, Veterinearplatz 1, 1210 Vienna, Austria

**Keywords:** domestic dogs, gaze-following into distant space, object-choice task, ostensive cues, dog–human communication

## Abstract

Dogs are renowned for being skilful at using human-given communicative cues such as pointing. Results are contradictory, however, when it comes to dogs' following human gaze, probably due to methodological discrepancies. Here we investigated whether dogs follow human gaze to one of two food locations better than into distant space even after comparable pre-training. In Experiments 1 and 2, the gazing direction of dogs was recorded in a gaze-following into distant space and in an object-choice task where no choice was allowed, in order to allow a direct comparison between tasks, varying the ostensive nature of the gazes. We found that dogs only followed repeated ostensive human gaze into distant space, whereas they followed all gaze cues in the object-choice task. Dogs followed human gaze better in the object-choice task than when there was no obvious target to look at. In Experiment 3, dogs were tested in another object-choice task and were allowed to approach a container. Ostensive cues facilitated the dogs’ following gaze with gaze as well as their choices: we found that dogs in the ostensive group chose the indicated container at chance level, whereas they avoided this container in the non-ostensive group. We propose that dogs may perceive the object-choice task as a competition over food and may interpret non-ostensive gaze as an intentional cue that indicates the experimenter's interest in the food location she has looked at. Whether ostensive cues simply mitigate the competitive perception of this situation or they alter how dogs interpret communicative gaze needs further investigation. Our findings also show that following gaze with one's gaze and actually choosing one of the two containers in an object-choice task need to be considered as different variables. The present study clarifies a number of questions related to gaze-following in dogs and adds to a growing body of evidence showing that human ostensive cues can strongly modify dog behaviour.

## 1. Introduction

Communication is defined as the transmission of information (signal) between a signaller and a receiver, with both partners benefiting from the interaction [1]. Humans are extremely good at communicating with each other even in the absence of extended language [2]. During social interactions, humans mostly communicate ostensively, that is, they use behaviours to address their partner and to express their communicative intent [2,3]. When an individual A (sender) ostensively communicates about a visual target with another individual B (receiver), A's communication consists of two parts: communicating to B the intent to show the target (ostensive part) and showing the target (referential part) [3,4]. The ostensive signals used during such communication serve to unambiguously specify that the receiver is the addressee of the communicative act and to induce preferential orientation towards the sender [5,6]. A direct gaze that generates eye contact and calling the receiver's name have been proposed to be effective ostensive signals [3,6–8]. After the receiver's attention has been called, by means of referential signals the sender can then direct the attention of the receiver to a target, i.e. a third person, an object or an event [9]. This can be achieved by using global body orientation, deictic and iconic manual gestures, such as pointing, more subtle forms of visual orientation such as turning the face or only the eyes to a certain direction, and other attention-directing tactile or auditory signals [9,10].

In contrast to a broad range of animal species that readily follow not only conspecific but also human gaze into distant space even if the subject's attention has not been called before the sender looks to a certain direction (e.g. [11–13]), human infants at an early age follow gaze only if the directional gaze cue has been accompanied by ostensive cues [14–16]. However, it has recently been shown that also dogs’ following of human-given referential cues depends on the use of ostensive signals by the human demonstrator [17,18]. Dogs are extremely sensitive to human communicative signals and seem to react to them similarly to human infants [19,20]. Regarding the ostensive component of communication, dogs do not only respond to communication about an object differently after the human has addressed them by establishing eye contact and calling their name, but they also preferentially respond to dog-directed speech (with, e.g. heightened pitch) [21]. In regard to the referential component of communication, dogs, similarly to 2.5-year-old children, follow not only human gestures, such as pointing, to locate objects but also cues that humans hardly use during everyday life [22–24].

Despite this outstanding performance of dogs in using human cues, when it comes to following gaze, results on dogs are so far not as clear. Dogs' ability to follow human gaze has been tested in two different paradigms. First, in the gaze-following into distant space task, the experimental context provides no *a priori* information what another individual would be likely to look at; still, following its gaze into its general environment may help to detect a visual target such as food, a potential predator or an interesting social event. Second, the so-called object-choice task tests whether the subject follows the demonstrator's gaze to one of two potential food locations. The two tasks differ in various methodological details that may affect dogs' gaze-following behaviour.

When considering gaze-following into distant space, albeit pack-living dogs spontaneously follow their packmates’ gaze under naturalistic conditions [25], neither they nor pet dogs follow human gaze into distant space in experimental set-ups [25–27]. So far, one single study has found that pet dogs reliably follow human gaze, but the experimental paradigm used differed in two aspects from the classical methods [28]. First, the experimenter called the subjects' attention and made a distinctively surprised facial expression before producing the gaze cue, and, second, she looked at the door of the room where the experiment was conducted, that is, unlike in classical distant space tasks, there was a rather clear target of her gaze cue.

In contrast to the mostly negative results of the distant space experiments, there is a wide spread belief that dogs readily follow human gaze in object-choice tasks [19,29]. However, most studies that produced such results tested dogs on gaze-following after a more or less intensive training with easier social cues, such as pointing, bowing and touching [26,30–32]. Moreover, in some studies, the demonstrator actually combined her/his gaze with a more conspicuous pointing or tapping cue [33,34]. By contrast, in studies where dogs were tested without training for their spontaneous use of gaze cues, the dogs mostly failed to follow human gaze (in the sense that they approached one of the two containers randomly) or even avoided the gazed-at container [17,32,35]. Whether and how the experimenter addressed the dog before delivering her gaze cue varied across and within these studies. To date, only one study found that dogs spontaneously followed human gaze in an object-choice task [36]. Importantly, in this study, dogs only succeeded if the gaze cue had been given repeatedly and in a communicative manner, after attracting the dogs’ attention to the demonstrator by calling their names. Further, in this study, it was only coded whether the dogs looked at the container the demonstrator had looked at; they were not requested to make an actual choice of the two containers by approaching one of them.

Because of all the procedural differences, it is difficult to compare whether and when dogs follow human gaze into distant space or to one of the multiple objects. In fact, one single study exists that systematically compared the performance of dogs in both situations, using the same gaze cue [26]. This direct comparison found that dogs followed human gaze to locate hidden food but not into distant space. Unfortunately, also in this study, the critical trials in the object-choice task were preceded by training with other cues, which was not the case in the distant space task. Therefore, in the current study, we set out to investigate in a rigorous manner whether dogs, even without pre-training that could influence their performance, indeed follow gaze more in an object-choice situation than into distant space. Our prediction was that, due to the presence of the food locations one of which is the clearly defined target of the gaze cue, dogs would follow the human gaze more in the object-choice than in the distant space task. Second, in both situations, we used three different kinds of gaze cues in order to investigate whether repeated cuing and adding ostensive cues to the gaze cue would improve gaze-following in dogs. We predicted that, in both tasks, dogs would perform better after having been addressed and that administering the gaze cue repeatedly would further improve their performance. We indeed predicted that the communicative context should induce an expectation of finding something relevant at the cued location.

## 2. Experiment 1: gaze-following into distant space and in an object-choice task

### 2.1. Material and methods

#### 2.1.1. Subjects

We tested 65 pet dogs of various breeds (41 females and 24 males), all naive for gaze-following and object-choice experiments. They were all between 1 and 12 years of age (average: 5.1 years old) and had not shown noticeable signs of ageing. Pet dogs and their owners were recruited from the Clever Dog Lab database and participated on a voluntary basis. See the electronic supplementary material, table S1 for more details on dogs participating in Experiments 1 and 2.

#### 2.1.2. Experimental setting

The study took place at the Clever Dog Lab, Messerli Research Institute, University of Veterinary Medicine, Vienna, in Austria, from January to June 2013. Testing sessions were conducted in an enclosed and quiet room (7.25 m× 6.05 m). The owners were present in the room and handled their dog during the experiments. The experimenter (C.D.) was the human demonstrator in both experiments. Each dog had its usual collar or harness, but all had the same leash—supplied by the experimenter—to not have different lengths or weights. The leash was held by the owner very slackly so that it could not influence the dogs' reactions (head movements or choice). All trials were video-recorded with four cameras placed in four corners of the room and analysed later on.

#### 2.1.3. Procedure

Before the beginning of the experiment, dogs were released to explore the testing room for 5 min to get familiar with it and the experimenter. The experimenter always interacted in a kind and positive way with the dogs to make them feel comfortable. Each dog was tested in six trials: three trials in the distant space and three trials in the object-choice task. The owner was blindfolded during trials to avoid any potential cue they could have provided to the dog.

#### 2.1.4. Condition 1: gaze-following into distant space

Here we tested if pet dogs follow the three different kinds of gaze cues of a human demonstrator into distant space; that is, the dogs were tested in an empty room where the context was set up so that it did not define where the experimenter would look.

*Warm-up:* Before the experiment, the experimenter walked around in the room randomly and gave some pieces of food to the dog to make sure that the dog was interested in her.

**Figure 1.**
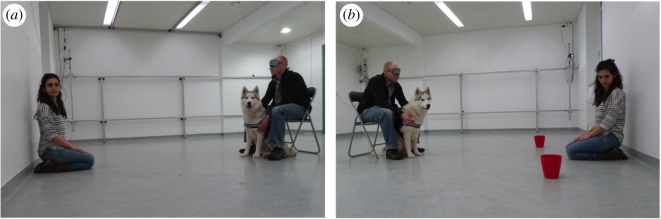
Experimental setting. (*a*) *Condition 1: gaze-following into distant space.* Subject dog is sitting and kept on slack leash by the owner, who is sitting behind it. The experimenter, kneeling opposite the dog, looks to one side of the empty room. (*b*) *Condition 2: gaze-following in an object-choice task.* Subject dog is sitting and kept on slack leash by the owner, who is sitting behind it. The experimenter, kneeling opposite the dog, looks at one of the two containers (that are empty at this moment).

*Experimental trials:* The subject dog was sitting and kept on leash by its owner who was sitting behind it on a chair. The experimenter was kneeling 2.5 m in front of the dog, and in each of the three trials she looked either to the right or to the left (the direction of the gaze was the middle of the right or left wall)—her gaze direction was pseudo-randomized within each dog so that in two trials she looked in the same direction, whereas in one of the first, second or third trials she looked in the other direction (figure 1*a*). Her looking direction in each of the three trials was counterbalanced across dogs.

Each dog was tested in three trials: (i) *Single non-ostensive trial:* the experimenter waited for the dog to look at her and to establish eye contact and then made a single sudden look to the predetermined direction for approximately 5 s. (ii) *Single ostensive trial*: the experimenter lifted her eyebrows, called the dog ‘*dog's name*, look!’ and then, as soon as eye contact was established, made a single sudden look to the predetermined direction for approximately 5 s. (iii) *Repetitive ostensive trial*: the experimenter lifted her eyebrows, called the dog ‘*dog's name*, look!’, and then, as soon as eye contact was established, made three consecutive sudden looks to the predetermined direction within approximately 5 s. Before each looking, she addressed the dog in the same way. Each trial ended 10 s after the last gaze cue and was followed by a break. During the 10-min long breaks, the experimenter interacted in a positive way with dogs (petting and playing) and then gave them a treat as a reward to keep them interested in her. The order of trials was counterbalanced across dogs.

#### 2.1.5. Condition 2: gaze-following in an object-choice task

In Condition 2, we tested if pet dogs would follow the three kinds of gaze cues of the human demonstrator in an object-choice task, that is after warm-up trials that informed them that they can find food in one of the two containers placed in the room. Importantly, however, unlike in most other object-choice experiments, our warm-up did not allow for direct associations between these food locations and the human demonstrator. Further, because our aim was to directly compare the dogs' performance in this test and in Condition 1, in contrast to most other object-choice tasks, we did not let the dogs approach a container after the gaze cue, but simply coded whether they followed the experimenter's gaze with their own gaze. This again prevented the dogs from associating the experimenter's gaze cue with food.

*Warm-up:* At the beginning of this condition, two single non-social warm-up trials were conducted: two containers with food in both of them were placed at random locations in the room. The dog was released into the room and allowed to explore the containers and to eat the food. If the dog did not explore the containers by itself, the owner could encourage it. The aim was to let the dogs learn that the containers contain food.

*Experimental trials:* The dog was sitting and held on leash by its owner who was sitting behind it on a chair. At the beginning of each trial, the experimenter went to her predefined place 2.5 m in front of the dog and put both empty containers on her two sides, 1.2 m from each other (figure 1*b*). In this way, both containers were at a distance of 2 m from the dog. In each of the three trials, the experimenter looked at one of the two containers with the side pseudo-randomized in the same way as in Condition 1.

Each dog was tested in three trials: (i) *Single non-ostensive trial:* the experimenter waited for the dog to look at her and to establish eye contact and then made a single sudden look to the predetermined container (left or right) for approximately 5 s. (ii) *Single ostensive trial*: the experimenter lifted her eyebrows, called the dog ‘*dog's name*, look!’ and then, as soon as eye contact was established, made a single sudden look to the predetermined container for approximately 5 s. (iii) *Repetitive ostensive trial*: the experimenter lifted her eyebrows, called the dog ‘*dog's name*, look!’, and then, as soon as eye contact was established, made three consecutive sudden looks to the predetermined container within approximately 5 s. Before each look, she addressed the dog in the same way. Each trial ended 10 s after the last gaze cue and was followed by a break. During each break, dogs did one warm-up trial, with food hidden in only one of the containers to keep them interested.

#### 2.1.6. Data collection

For both Conditions 1 and 2, in each trial, we recorded whether and how often the dogs looked (i.e. they visibly turned their head) in the direction indicated by the experimenter within 10 s after the gaze cue, and whether their first look to the side was into this direction or not. All trials were analysed by the experimenter (C.D.) from the video records using Solomon Coder (copyright by András Péter, http://solomoncoder.com/). Approximately 20% of the trials were coded for reliability by an uninformed experimenter, Geri Werhahn, and excellent consistency was found: (i) first look in gaze cue direction: Cohen's *κ* = 0.90, 95% CI = (0.62–0.98), *p* < 0.01; (ii) number of looks in gaze cue direction: Spearman's correlation *r* = 0.85, *p* < 0.001.

#### 2.1.7. Statistical analysis

To compare the behaviour of the dogs across the two conditions and across the three trials, and also to examine whether age in months, sex and gaze direction (left versus right) influenced their performance, we calculated generalized linear mixed models (glmm), using software R. 2.15.3. The individual's identity was included as a random factor. Depending on the exact variable analysed, we used a glmm either with binomial or with Poisson distribution. When necessary, we corrected for multiple tests with Holm–Bonferroni's correction. Further, to investigate whether more dogs than expected by chance (because only two directions (condition 1) or targets (condition 2) were present, we expected the dogs to look either at the one or the other direction/target, setting the chance level at 50%) followed the gaze cue with their first look we used a one-sample *t*-test.

### 2.2. Results

An overview of the main results is provided in table 1.

**Table 1.** Overview of results of Experiment 1. ‘Condition’ refers to the comparison between Condition 1 (gaze-following into distant space) and Condition 2 (gaze-following in the object-choice task). More details on the significant effects are provided in the text. ‘Trial’ refers to the comparison between single non-ostensive, single ostensive and repetitive ostensive trials. When a trial had a significant effect, more details on the pairwise statistical comparisons of the trials are provided in the text. Italics emphasize significant results.

dependent variables	independent variables	*p*-value
Did the dogs look to a side or remain watching the experimenter for 10 s?	*Condition*	*<0*.*001*
	*Trial*	>0.05
If dogs looked, did they follow the experimenter's gaze at least once?	*Condition*	*0*.*036*
	*Trial*	*<0*.*05*
If dogs looked to a side, was their first look to the gaze cue direction?	*Condition*	*0*.*024*
	*Trial*	*<0*.*05*
Numbers of looks in the gaze cue direction in those trials when there was at least one such look	Condition * Trial	*0*.*036*

When tested with gaze into distant space, dogs were less likely to look to either side (*N* = 22 dogs stayed gazing at the experimenter in all of their three trials) than when tested with the two pots (*N* = 5 dogs stayed facing the experimenter) (glmm with binomial distribution: *F*_1,324_ = 43.000, *p* < 0.001). However, we found no influence of trial, age, sex, direction of gaze cue and no significant interaction between condition and trial (glmm: trial: *F*_2,322_ = 0.64, *p* = 0.50; condition *× *trial: *F*_1,319_ = 0.23, *p* = 0.80; age: *F*_1,63_ = 2.40, *p* = 0.10; sex: *F*_1,62_ = 0.33, *p* = 0.60; direction of gaze cue: *F*_1,327_ = 0.20, *p* = 0.70).

Analysing whether the dogs looked in the indicated direction (out of those trials in which they did look to a side), we found that in the distant space task, the dogs were less likely to look to the indicated side than when tested with the two containers (glmm: *z* = −2.097, *p* = 0.036). We found no significant interaction between the condition and the trial (glmm: *z* = 1.18, *p* = 0.86). Furthermore, in both contexts, dogs were less likely to look in the indicated direction in the non-ostensive trial than in the repetitive ostensive (glmm: *z* = −2.300, *p* = 0.021) and the single ostensive (glmm: *z* = −2.02, *p* = 0.04) trials. However, the repetitive ostensive and the single ostensive trials did not differ from each other (glmm: *z* = −1.48, *p* = 0.12).

**Figure 2.**
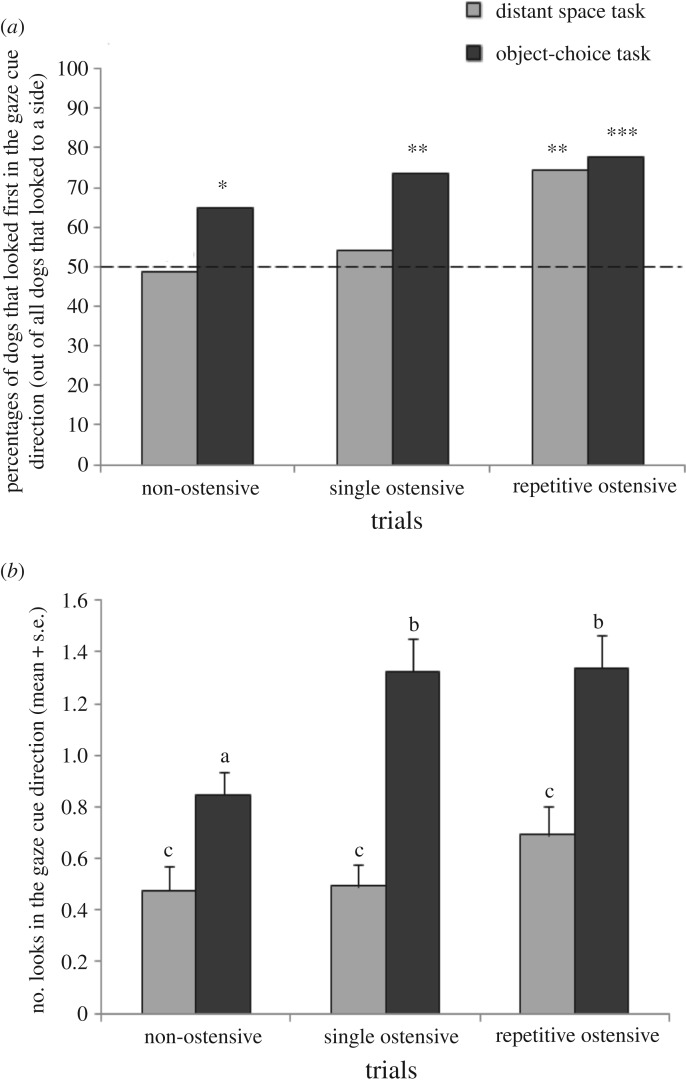
(*a*) Percentages of dogs that looked first in the gaze cue direction in Conditions 1 and 2 out of all dogs that looked to a side within 10 s after the gaze cue. Grey bars correspond to Condition 1 (gaze-following into distant space) and black bars correspond to Condition 2 (gaze-following in the object-choice task); the 50% line represents chance level. Asterisks indicate significant differences from chance level obtained with *t*-tests. *p < 0.05; **p < 0.01; ***p < 0.001. (*b*) Number of looks following the gaze cue in Conditions 1 and 2 (mean + s.e.). Grey bars correspond to Condition 1 (gaze-following into distant space) and black bars correspond to Condition 2 (gaze-following in the object-choice task). Different letters (a, b, c) indicate significant differences between conditions (glmm).

Furthermore, we investigated in each trial how many dogs looked first into the gaze cue direction demonstrated by the experimenter and whether this number was higher than expected by chance. As for the previous variable, dogs that did not look to the side but remained watching the experimenter were excluded from these analyses (number of dogs that remained in the analyses: into distant space, *N*_non–ostensive_ = 39, *N*_single ostensive_ = 37, *N*_repetitive ostensive_ = 39; in the object-choice task, *N*_non–ostensive_ = 54, *N*_single ostensive_ = 53, *N*_repetitive ostensive_ = 52). In the distant space task, the dogs did not follow the gaze cue significantly above chance level with their first look in the non-ostensive (48.7%, *t*-test: *t*_76_ = 0.22, *p* = 0.82) and in the single ostensive trials (54%, *t*-test: *t*_72_ = 0.23, *p* = 0.82; figure 2*a*). However, in the repetitive ostensive trial, significantly more dogs than expected by chance followed the gaze cue with their first look (74%, *t*-test: *t*_76_ = 2.38, *p* = 0.01; figure 2*a*). In the object-choice task, we found that the dogs looked, with their first look, into the indicated direction significantly above chance level in all trials (*t*-tests; single non-ostensive trial: 64.8%, *t*_106_ = 1.98, *p* = 0.05; single ostensive trial: 73.6%, *t*_104_ = 2.45, *p* = 0.01; repetitive ostensive trial: 77.6%, *t*_114_ = 3.19, *p* = 0.001; figure 2*a*).

Confirming these findings, the dogs were less likely to look first to the indicated direction when tested in the distant space task than when tested with the two containers (glmm: *z* = 2.256, *p* = 0.024). There was no significant interaction between the condition and the trial (glmm: *z* = 1.127, *p* = 0.57). Furthermore, in both conditions, the dogs were less likely to look first into the indicated direction in the non-ostensive trial than in the repetitive ostensive trial (glmm: *z* = 2.664, *p* = 0.007), but we found no difference either between the non-ostensive and the single ostensive trials (glmm: *z* = 1.040, *p* = 0.298) or between the single ostensive and the repetitive ostensive trials (glmm: *z* = 1.625, *p* = 0.10). In addition, we found that if the cue was given to the right side, then the dogs were more likely to look first in the indicated direction than when it was given to the left side (glmm: *z* = −2.099, *p* = 0.036). The other factors did not influence the first gaze direction of the dogs (glmm: age: *z* = 0.07, *p* = 0.94; sex: *z* = −0.28, *p* = 0.78).

Analysing the total number of looks to the indicated side, we took only those trials into account when at least one such look occurred. We found an interaction between the condition and the trial (glmm: *F*_2,231_ = 3.370, *p* = 0.036). While we found no influence of trial when tested with the gaze into distant space (glmm: *F*_2,85_ = 2.19, *p* = 0.10), we did so when looking at one of the two containers (*F*_2,113_ = 8.350, *p* < 0.001; figure 2*b*). The dogs looked less often at the indicated pot when tested in the non-ostensive trial than when tested in the other two (glmm: non-ostensive versus repetitive ostensive: *z* = 2.247, *p* = 0.025; non-ostensive versus single ostensive: *z* = 2.697, *p* = 0.007; figure 2*b*). Furthermore, we found an age effect (glmm: *F*_1,66_ = 5.080, *p* = 0.028). With increasing age, the number of looks to the indicated side decreased. There was no influence of sex and direction of gaze cue (glmm, respectively, *F*_1,62_ = 0.49, *p* = 0.48; *F*_1,233_ = 3.72, *p* = 0.06).

### 2.3. Discussion for Experiment 1

In summary, we found that the dogs were more likely to look to either side in the object-choice task than in the distant space task in which they more often remained watching the experimenter after the gaze cue. More interestingly, we also found that in those trials when the dogs did look to a side, they followed the experimenter's gaze cue more often in the object-choice than in the distant space task. Despite this overall difference between the two conditions, in both tasks the ostensiveness of the gaze cue influenced whether the first look of the dogs was to the indicated direction or not. In both conditions, the dogs looked first into the demonstrated direction more often after the repetitive ostensive gaze cue than in the non-ostensive trial. This effect was more pronounced in the distant space task where the dogs reliably followed the gaze cue with their first look only in the repetitive ostensive condition, in contrast to the object-choice task where the dogs looked first at the indicated pot in all three trials; however, they did so more often the more ostensive the cues were.

These results suggest three key findings. First, dogs followed human gaze into distant space given that the demonstrator used repeated ostensive gaze shifts. Previously, two studies had shown that dogs follow human gaze geometrically [27,28]. These studies, however, albeit they tested the dogs outside an object-choice task, did offer a clear visual target for the experimenter's gaze: in one of them [28], the experimenter looked at the door of the room, and in the other [27], at some food placed on one side of a barrier. This might have made gaze-following more likely in these two tasks than in a classic distant space task. Relevant for the latter, two former studies had tested whether dogs follow human gaze into distant space and both found negative results [25,26]. This is, however, in line with our findings, because both of these studies used a single gaze cue with minimal communication.

Second, our study confirms that dogs indeed follow human gaze more in a two-way object-choice task than into distant space. This difference is probably due to the fact that in the object-choice task, the demonstrator looked in the direction of a potential food container. This result may mean that dogs, similarly to human infants [37], better/preferentially follow referential gaze, that is gaze cues that are congruent with object locations. Alternatively or additionally, the fact that the object-choice task is a foraging task where dogs expect to find food may increase the dogs' motivation to follow gaze and/or to pay attention to the demonstrator [27]. We encourage further study to control for the foraging context by conducting trials with pots and no-pot controls in both conditions.

Third, we confirmed previous findings that dogs better follow human gaze when it is accompanied by ostensive communicative cues [36]. Our study adds to former findings in showing that this is so not only in object choice but also in a distance space task. More precisely, our Experiment 1 has shown that extensive communication (eye contact with *repetitive* calls and gaze shifts) is actually necessary to trigger reliable gaze-following into distant space in pet dogs.

Experiment 1 still has some limitations. First, even if our results emphasize the necessity of communication for eliciting dogs’ responses, it is possible that dogs better followed gaze in the ostensive context because they were not paying enough attention in the non-ostensive trial. It is also possible that not communication but repetition is the key to making the gaze cue in our study more conspicuous for dogs. Our protocol does not allow us to distinguish between the effects of ostensive cues and the repetition of the gaze cue itself, as we did not test the dogs in a repetitive non-ostensive condition. Second, in Condition 2, the dogs were not allowed to make a choice between the two pots, which makes comparisons with other relevant studies [17] difficult. We thus decided to run a second experiment to address these issues.

## 3. Experiment 2: gaze-following and choice in an object-choice task

In Experiment 2, we tested dogs in another object-choice task where they were actually allowed to make a choice by approaching one of the two containers, after having received either repeated ostensive or non-ostensive gaze cues. We thus wished to verify if following with the gaze is indicative of choosing the indicated container, in a protocol comparable with previous studies.

Since one can argue that dogs follow ostensive communicative cues better because they pay more attention to them, in Experiment 2 a non-ostensive attention-caller preceded each gaze cue in the non-ostensive condition. In both conditions and all trials, the owners were blindfolded to avoid any potential cue they could have provided to the dogs.

### 3.1. Material and methods

#### 3.1.1. Subjects

We tested 38 pet dogs of various breeds (22 females and16 males). They were all between 1 and 11.5 years old (average: 4.8 years old) and had not shown noticeable signs of ageing. Pet dogs and their owners were recruited from the Clever Dog Lab database and participated on a voluntary basis. Each dog was tested in 10 trials, in one of the two conditions (with repetitive non-ostensive (*N* = 19) or repetitive ostensive gaze (*N* = 19); see electronic supplementary material, table S1 for more details).

#### 3.1.2. Experimental set-up

**Figure 3.**
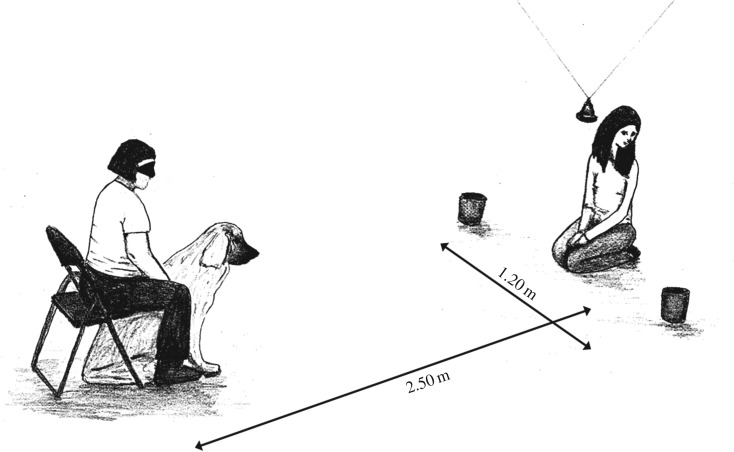
Experimental setting of Experiment 2. The experimental set-up is the same as in Experiment 1 Condition 2 with the only addition being that a bell is hanging from the ceiling high enough to let the dog see the experimenter's face. In the non-ostensive group, the experimenter rings this bell in order to call the dog's attention.

The subject dog was sitting and held on leash by its owner who was sitting behind it on a chair. The experimenter was 2.5 m in front of the dog, with both containers on her two sides, 1.2 m from each other. In this way, both containers were 2 m distant from the dog. The experimenter was kneeling under a bell, suspended from the ceiling, high enough for the dog to see the face of the experimenter (figure 3).

#### 3.1.3. Procedure

*Pre-training:* Before the experiment began, all dogs participated in the pre-training in order to ensure that they were not scared of the bell and they paid attention to the experimenter calling their attention either by calling their name or by ringing the bell. The owner was sitting on a chair, holding her/his dog between her/his legs. The experimenter was kneeling in front of the dog, under the bell. Then, the experimenter either rang the bell by moving it with her nose or called the dog, and the dog was rewarded with a piece of food when looking at the experimenter (the experimenter threw a piece of food to the dog). When the dog looked at the experimenter reliably upon being called or ringed at (that is, reacted immediately to three consecutive calls or bell-rings), the pre-training ended. This was typically achieved in less than 10 min. At this point, the owner left the room with the dog for a 10 min break after which the experiment began.

*Warm-up:* Four non-social warm-up trials were conducted in the same way as in Condition 2 of Experiment 1.

*Experimental trials:* The subject dog was sitting and kept on leash by its owner who was sitting behind it on his/her assigned chair. The experimenter went to her place in front of the dog and placed the two empty containers on her two sides. Each dog was tested in one of the two following conditions: (i) *Repetitive non-ostensive gaze:* the experimenter rang the bell with her nose to call the dog's attention. As soon as the dog looked at the experimenter, she looked at the predetermined container (sides were randomized and counterbalanced within and across dogs). Calling the dog's attention with the bell and looking at the predefined container were repeated three times within approximately 5 s in each trial. (ii) *Repetitive ostensive trial*: the experimenter lifted her eyebrows, called the dog ‘*dog's name*, look!’, and then, as soon as eye contact was established, looked three times at the predetermined container within approximately 5 s, calling the dog's attention each time.

After the repeated cuing, the owner released the dog that was then free to go and choose a container. Each dog was tested in a single session of 10 trials.

#### 3.1.4. Data collection

In each trial, we recorded whether and how often the dogs looked in the direction indicated by the experimenter within 5 s after the gaze cue, and whether their first look to a container was towards the baited or the empty one. We also recorded whether and which pot the dogs chose. A choice was defined as approaching a container and inserting one's nose in it within 10 s after being released.

All trials were analysed by the experimenter (C.D.) from the video records using Solomon Coder (copyright by András Péter, http://solomoncoder.com/). Approximately 20% of the trials were coded for reliability by an uninformed experimenter, and excellent consistency was found: (i) first look in gaze cue direction (yes/no): Cohen's *κ* = 0.96, 95% CI = (0.93–0.99), *p* < 0.01; (ii) number of looks in gaze cue direction: Spearman's correlation *r* = 0.9, *p* < 0.001; and (iii) number of correct choices: Spearman's correlation *r* = 1.00.

#### 3.1.5. Statistical analysis

To compare dogs’ behaviour in the two conditions (ostensive versus non-ostensive), and also to examine whether age in months, sex and demonstrated gaze direction influenced their performance, we calculated glmm with these four fixed factors, using software R. v. 2.15.3. The individuals' identity was included as a random factor. We used a glmm with binomial distribution for the variables *first look in gaze cue direction* and *choice of the indicated pot*, and a glmm with Poisson distribution for the variable *number of looks in gaze cue direction*. We compared the variables *first look in gaze cue direction* and *choice of the indicated pot* with the chance level for all 10 trials using a one-sample *t*-test.

### 3.2. Results

#### 3.2.1. Following gaze with gaze

**Figure 4.**
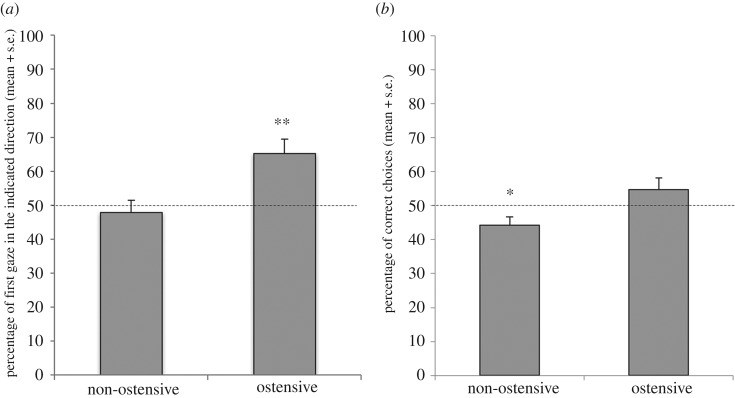
(*a*) Percentage of first gaze in the indicated direction in 10 trials in Experiment 2. Dogs performed above chance level in the ostensive group. (*b*) Percentage of correct choices in 10 trials in Experiment 2. Dogs performed below chance level in the non-ostensive group; 50% line represents chance level. Asterisks indicate performances significantly different from chance, *p < 0.05; **p < 0.01.

All dogs in all trials looked at least once to the indicated container, but the dogs in the ostensive condition were more likely to look first at the indicated pot than in the non-ostensive condition (glmm: *z* = 3.387, *p* = 0.001). Moreover, while we found no influence of sex on the probability to look first at the indicated container (glmm: *z* = 0.004, *p* = 0.997), older dogs were more likely to do so than younger dogs (glmm: *z* = 2.013, *p* = 0.044). Looking at the conditions separately, we found that dogs followed the demonstrator's gaze at chance level in the non-ostensive condition (*t*-test: *t*_36_ = 0.58, *p* = 0.566), whereas they did so above chance in the ostensive condition (*t*-test: *t*_36_ = 3.62, *p* < 0.001, figure 4*a*).

We also found that dogs in the ostensive condition looked more often at the indicated pot than those in the non-ostensive condition (glmm: *F*_1,36_ = 9.440, *p* = 0.004), while neither age nor sex had any influence (glmm: sex: *F*_1,35_ = 0.06, *p* = 0.80; age: *F*_1,34_ = 0.03, *p* = 0.86).

Finally, we controlled and did not find any influence of order of trials on dogs' first looking behaviour (glmm: *z* = 2.143, *p* = 0.143).

#### 3.2.2. Choices

Moreover, the dogs were more likely to choose the indicated pot in the ostensive than in the non-ostensive condition (glmm: *F*_1,378_ = 4.240, *p* = 0.040). Neither age nor sex influenced the dogs’ number of correct choices (glmm: sex: *F*_1,376_ = 0.03, *p* = 0.85; age: *F*_1,377_ = 2.02, *p* = 0.16). Dogs performed at chance level in the ostensive condition (one-sample *t*-test: *t*_18_ = 1.41, *p* = 0.18), whereas their performance was below chance level in the non-ostensive condition (one-sample *t*-test: *t*_18_ = −2.357, *p* = 0.030; figure 4*b*).

Finally, we controlled and did not find any effect of trial order on dog's correct choice (glmm: *z* = 0.005, *p* = 0.934).

### 3.3. Discussion for Experiment 2

The results of this experiment confirmed that whether or not human gaze is accompanied by ostensive cues influences the behaviour of dogs in an object-choice task. Only in the ostensive condition did dogs follow the experimenter's gaze with their first look to the indicated pot above chance level. Additionally, in this condition, the dogs looked to the indicated direction more often than in the non-ostensive condition. Interestingly, even though the dogs followed the human's gaze to the baited container in the ostensive condition, they chose the indicated container only at chance level. By contrast, dogs in the non-ostensive conditon actually avoided the container.

Our results confirm that dogs better follow ostensive than non-ostensive human gaze, and, importantly, they do so not because of a lack of attention in the non-ostensive context. Still, the gazing behaviour of the dogs in the non-ostensive condition differed from that of the dogs tested in Experiment 1 Condition 2 in the non-ostensive trial. Whereas in Experiment 1, the first look of most dogs was directed at the indicated container, in Experiment 2 the dogs showed no such preference. It is possible that in Experiment 2, the training to look toward the bell and the experimenter reduced the dogs’ gaze-following response, as a former study has shown that training to look at humans decreases gaze-following in dogs [28].

Importantly, in Experiment 2, we coded two variables: following gaze with gaze and choice. We found that these two measures provide different results. Interestingly, similarly to Experiment 1, dogs followed ostensive human gaze with their gaze in this object-choice task, but when they were released to choose a container, they did not preferentially approach the indicated container but chose randomly. These data confirm previous findings: without previous training, dogs do not choose the indicated container, even if it had been indicated with repetitive ostensive gaze [17]. Also in the non-ostensive context, the two kinds of gaze-following responses brought different results: the dogs looked at one of the two containers randomly but then avoided the baited container in the choice phase. Our choice data in both groups contradict the suggestion that dogs interpret human gaze as a cooperative signal. It has been proposed that dogs outperform chimpanzees in locating hidden food based on human-given cues because they better understand the cooperative, food-sharing message of pointing and gazing than chimpanzees, who use such cues more in competitive contexts (*Cooperative Communicative Gaze Hypothesis* [29,38,39]). In line with our results, also former studies had found that dogs avoid a food location a human or a conspecific had looked at beforehand [32,35,40]. Based on findings that dogs avoid the food location someone else looked at beforehand, we propose that dogs may interpret gaze as an intentional cue, or at least as a behavioural cue that reliably indicates others' further actions (*Intentional Gaze Cue Hypothesis*). That is, dogs may use this behavioural cue to predict that the next action of their partner watching a food container will be to approach this location and to try to get the food herself/himself. A number of studies have shown that both human and non-human primates perceive gaze (head direction) as an intentional cue and use the information to predict what others will do next [41]. When an actor is facing two objects, 12-month-old infants [42], chimpanzees [43] or even tamarins [44] can identify which object the actor is likely to grasp based on their gaze direction. In non-human primates, these results are especially profound in competitive contexts [43]. If dogs, as suggested above, consider the object-choice task as possible food competition and they are shy or not very self-confident or simply prefer to avoid a potential conflict, they can be expected to avoid the food container their partner has looked at in a non-ostensive manner. If they still want to attempt to find food avoiding the indicated container, they will go to the other food location. Such an interpretation of non-ostensive gaze may also explain why in the non-ostensive condition of Experiment 2 the dogs looked less often at the baited container than in the non-ostensive trial of Experiment 1 Condition 2. The repeated non-ostensive gaze cue applied in Experiment 2 may be seen as a stronger intentional cue, and therefore as a stronger indication of a potential conflict, than the single, non-ostensive gaze cue of Experiment 1.

## 4. General discussion

In summary, our study confirmed our predictions: the dogs followed human gaze to objects easier than into distant space, and the results emphasized the role of ostensive communication in facilitating gaze-following. Our study also clearly shows that following human gaze by looking at an object and physically approaching a container (choice) in the object-choice task need to be considered as independent and different variables.

As former studies have also demonstrated in various other contexts [17,18,45–47], our results clearly show that dogs respond differently to human behaviour accompanied or not by ostensive cues. Regarding the developmental origin of this differentiation, as pet dogs spend most of their time among humans and in an environment where humans pay attention to a lot of objects, persons and events few of which are relevant for dogs, they have plenty of opportunities to learn during their ontogeny which human cues can lead them to acquiring meaningful information [48–50]. This, on the one hand, may mean habituation and ceasing to respond to most of our gaze cues before which they are not addressed. On the other hand, dogs may also learn to pay increased attention to human behaviour after dog-directed communication and attention-calling [18,51].

While the importance of life experiences in dogs’ gaze-following response cannot be overemphasized, it may well be that such ontogenetic processes are influenced by genetic predispositions of dogs. Many have suggested that, due to their domestication, dogs are genetically prepared to learn about human cues [33,34,48,52,53], which makes learning about ostensive and non-ostensive human behaviours especially fast and effective. Alternatively, however, based on the currently available data, we cannot exclude that this fast learning in dogs does not rely on a specific adaptation to human ostension but rather on a capability that may support intraspecific communication in wolves [25]. In various social species, it has been shown that animals can learn which emotional facial expressions and other behavioural cues of their conspecifics indicate when their partners' gaze cues are relevant for them [54,55]. Such a skill, if present in wolves and dogs, may support learning about humans’ ostensive cues as well if dogs happen to share their life with human partners.

Independently from its evolution and development, what kind of mechanisms underlie the differential responding of dogs to ostensive and non-ostensive human gaze remains an intriguing question. As discussed earlier, we argue that dogs may perceive the object-choice task as a competition over food and may interpret non-ostensive gaze as an intentional or behavioural cue that indicates the experimenter's interest in the food location she has looked at. Based on our results, however, dogs perceive ostensive communicative gaze in a different way. When taking into account only that dogs, with their gaze, followed ostensive gaze more than non-ostensive gaze in both experiments, one could argue that dogs simply pay more attention to ostensive than to non-ostensive gaze. Our choice data in Experiment 2, however, show that dogs also do pay enough attention to non-ostensive gaze, as they clearly adjusted their choices to this cue and avoided the container the experimenter had looked at. It is still possible, however, that dogs continue to interpret also ostensive gaze as an intentional cue and presenting the gaze cue in an ostensive and repetitive fashion does not do more than mitigate the competitive perception of the object-choice task. This might be enough to stop dogs avoiding the food location the experimenter has looked at and make them follow the ostensive gaze cue more often with their gaze.

A third possibility is that their human partner's ostensive cues truly alter how dogs interpret her behaviour (in this case her directional gaze cue), as has been suggested for human infants [8,56]. After having been addressed by a demonstrator, human infants and possibly also dogs switch to a ‘more receptive mode’ and readily follow and learn from her behaviour, in contrast to non-ostensive situations when they tend to critically re-evaluate what they have incidentally observed [46,56,57]. Regarding the adaptive value of such an effect of human ostensive cues on dog behaviour, a recent hypothesis has suggested that it can facilitate not only information acquisition but also behavioural synchronization between dogs and humans, which in turn helps to avoid conflicts and/or to co-act in terms of common actions without necessarily comprehending the causal structure of the collaborative interaction [20,58]. In regard to gaze-following, this hypothesis suggests that dogs, in contrast to perceiving non-ostensive gaze as an intentional cue, may interpret human ostensive gaze as an imperative that sends them to a certain location. The same interpretation of the human pointing gesture and a human hiding a desired object repeatedly at a certain location has been proposed [53,57,59]. Gaze, however, is likely to have a weaker discriminative as well as an imperative component than pointing or repeated manipulation of an object. This means that dogs in the end may not understand which direction they should go or that they are sent somewhere, and for this reason they choose the indicated container only at chance level despite following the experimenter's gaze with their gaze.

To sum up, our study showed that dogs are able to follow at least repeated and ostensive human gaze also into distant space. Still, they perform better in object-choice tasks, which does not mean, however, that dogs comprehend human gaze as a cooperative communicative signal. We propose that in object-choice tasks, dogs interpret non-communicative human gaze as an intentional cue that indicates the others' interest in food, over which the dogs hesitate to get into conflict. Whether communication simply mitigates the competitive perception of this foraging situation or dogs interpret communicative gaze in a different way requires further research. Finally and importantly, we emphasize that dogs following human gaze with their gaze or by approaching a target location (choice) can show very different results and should, therefore, not be used interchangeably.

## Supplementary Material

Table S1 and additional results

## Data accessibility

Two files (data of Experiment 1 and data of Experiment 2) are available from Open Science Framework at: https://osf.io/r5q3k/?view_only=48db000312f44b0d8ac41d0066b73fae.

## References

[1] Smith JM, Harper DGC 1995 Animal signals: models and terminology. J. Theor. Biol. 177, 305–311. (doi:10.1006/jtbi.1995.0248)

[2] Knap ML, Hall JA, Horgan TG. 2014 Nonverbal communication in human interaction, 8th edn Belmont, CA: Wadsworth.

[3] Gómez JC 1996 Ostensive behavior in great apes: the role of eye contact. In Reaching into thought: the minds of the great apes (eds Russon AE, Bard KA, Taykor Parker S), pp. 131–151. Cambridge, UK: Cambridge University Press.

[4] Sperber D, Wilson D. 1986 Relevance: communication and cognition. Oxford, UK: Basil Blackwell.

[5] Csibra G 2010 Recognizing communicative intentions in infancy. Mind Lang. 25, 141–168. (doi:10.1111/j.1468-0017.2009.01384.x)

[6] Leslie A M, Happé F 1989 Autism and ostensive communication: the relevance of metarepresentation. Dev. Psychopathol. 1, 205–212. (doi:10.1017/S0954579400000407)

[7] Cooper RP, Aslin RN 1990 Preference for infant-directed speech in the first month after birth. Child Dev. 61, 1584–1595. (doi:10.2307/1130766) 2245748

[8] Csibra G, Gergely G 2009 Natural pedagogy. Trends Cogn. Sci. 13, 148–153. (doi:10.1016/j.tics.2009.01.005) 19285912 10.1016/j.tics.2009.01.005

[9] Leavens DA, Hopkins WD, Thomas RK 2004 Referential communication by chimpanzees (*Pan troglodytes*). J. Comp. Psychol. 118, 48–57. (doi:10.1037/0735-7036.118.1.48) 15008672 10.1037/0735-7036.118.1.48

[10] Sodian B, Thoermer C 2004 Infant's understanding of looking, pointing, and reaching as cues to goal-directed action. J. Cogn. Dev. 5, 289–316. (doi:10.1207/s15327647jcd0503_1)

[11] Anderson JR, Mitchell RW 1999 Macaques but not lemurs co-orient visually with humans. Folia Primatol. 70, 17–22. (doi:10.1159/000021670) 10050063 10.1159/000021670

[12] Bugnyar T, Stöwe M, Heinrich B 2004 Ravens, *Corvus corax*, follow gaze direction of humans around obstacles. Proc. R. Soc. Lond. B 271, 1331–1336. (doi:10.1098/rspb.2004.2738) 10.1098/rspb.2004.2738 PMC1691735 15306330

[13] Range F, Virányi Z 2011 Development of gaze following abilities in wolves (*Canis lupus*). PLoS ONE 6, e16888 (doi:10.1371/journal.pone.0016888) 21373192 10.1371/journal.pone.0016888 PMC3044139

[14] D'Entremont B, Hains SMJ, Muir DW 1997 A demonstration of gaze following in 3- to 6-month-olds. Inf. Behav. Dev. 20, 569–572. (doi:10.1016/S0163-6383(97)90048-5)

[15] Farroni T, Massaccesi S, Pividori D, Johnson MH 2004 Gaze following in newborns. Infancy 5, 39–60. (doi:10.1207/s15327078in0501_2)

[16] Senju A, Csibra G 2008 Gaze following in human infants depends on communicatve signals. Curr. Biol. 18, 668–671. (doi:10.1016/j.cub.2008.03.059) 18439827 10.1016/j.cub.2008.03.059

[17] Kaminski J, Schulz L, Tomasello M 2012 How dogs know when communication is intended for them. Dev. Sci. 15, 222–232. (doi:10.1111/j.1467-7687.2011.01120.x) 22356178 10.1111/j.1467-7687.2011.01120.x

[18] Tauzin T, Csík A, Kis A, Topál J 2015 The order of ostensive and referential signals affects dog's responsiveness when interacting with a human. Anim. Cogn. 18, 975–979. (doi:10.1007/s10071-015-0857-1) 25771965 10.1007/s10071-015-0857-1

[19] Kaminski J, Nitzschner M 2013 Do dogs get the point? A review of dog–human communication ability. Learn. Motiv. 44, 294–302. (doi:10.1016/j.lmot.2013.05.001)

[20] Duranton C, Gaunet F 2015 Canis sensitivus: affiliation and dogs' sensitivity to others’ behaviour as the basis for synchronization with humans? J. Vet. Behav. Clin. App. Res. 10, 513–524. (doi:10.1016/j.jveb.2015.08.008)

[21] Burnham D, Kitamura C, Vollmer-Conna U 2002 What's new, pussycat? On talking to babies and animals. Science 296, 1435 (doi:10.1126/science.1069587) 12029126 10.1126/science.1069587

[22] Lakatos G, Gácsi M, Topál J, Miklósi Á 2012 Comprehension and utilisation of pointing gestures and gazing in dog–human communication in relatively complex situations. Anim. Cogn. 15, 201e213 (doi:10.1007/s10071-011-0446-x) 21927851 10.1007/s10071-011-0446-x

[23] Miklósi Á, Soproni K 2006 A comparative analysis of animals' understanding of the human pointing gesture. Anim. Cogn. 9, 81e93 (doi:10.1007/s10071-005-0008-1) 16235075 10.1007/s10071-005-0008-1

[24] Soproni K, Miklósi Á, Topál J, Csányi V. 2002 Dogs’ (*Canis familaris*) responsiveness to human pointing gestures. J. Comp. Psychol. 116, 27e34 (doi:10.1037/0735-7036.116.1.27) 11926681 10.1037/0735-7036.116.1.27

[25] Werhahn G, Virányi Z, Barrera G, Sommese A, Range F 2016 Wolves (*Canis lupus*) and dogs (*Canis familiaris*) differ in following human gaze into distant space but respond similar to their packmates' gaze. J. Comp. Psychol. 130, 288–298. (doi:10.1037/com0000036) 27244538 10.1037/com0000036 PMC5321535

[26] Agnetta B, Hare BA, Tomasello M 2000 Cues to food location that domestic dogs (*Canis familiaris*) of different ages do and do not use. Anim. Cogn. 3, 107–112. (doi:10.1007/s100710000070)

[27] Met A, Miklósi Á, Lakatos G 2014 Gaze-following behind barriers in domestic dogs. Anim. Cogn. 17, 1401–1405. (doi:10.1007/s10071-014-0754-z) 24816625 10.1007/s10071-014-0754-z

[28] Wallis LJ, Range F, Müller CA, Serisier S, Huber L, Virányi Z 2015 Training for eye contact modulates gaze following in dogs. Anim. Behav. 106, 27–35. (doi:10.1016/j.anbehav.2015.04.020) 26257403 10.1016/j.anbehav.2015.04.020 PMC4523690

[29] Hare B, Tomasello M 2005 Human-like social skills in dogs? Trends Cogn. Sci. 9, 339–444. 10.1016/j.tics.2005.07.003 16061417

[30] McKinley J, Sambrook TD 2000 Use of human-given cues by domestic dogs (*Canis familiaris*) and horses (*Equus caballus*). Anim. Cogn. 3, 13–22. (doi:10.1007/s100710050046)

[31] Miklósi Á, Polgárdi R, Topál J, Csányi V 1998 Use of experimenter-given cues in dogs. Anim. Cogn. 1, 113–121. (doi:10.1007/s100710050016) 24399275 10.1007/s100710050016

[32] Soproni K, Miklósi Á, Topál J, Csányi V 2001 Comprehension of human communicative signs in pet dogs (*Canis familiaris*). J. Comp. Psychol. 115, 122–126. (doi:10.1037/0735-7036.115.2.122) 11459158 10.1037/0735-7036.115.2.122

[33] Hare B, Tomasello M 1999 Domestic dogs (*Canis familiaris*) use human and conspecific social cues to locate hidden food. J. Comp. Psychol. 113, 173–177. (doi:10.1037/0735-7036.113.2.173)

[34] Hare B, Brown M, Williamson C, Tomasello M 2002 The domestication of social cognition in dogs. Science 298, 1634–1636. (doi:10.1126/science.1072702) 12446914 10.1126/science.1072702

[35] Oliva JL, Rault JL, Appleton B, Lill A 2015 Oxytocin enhances the appropriate use of human social cues by the domestic dog (*Canis familiaris*) in an object choice task. Anim. Cogn. 18, 767–775. (doi:10.1007/s10071-015-0843-7) 25647172 10.1007/s10071-015-0843-7

[36] Téglás E, Gergely A, Kupán K, Miklósi Á, Topál J 2012 Dogs’ gaze following is tuned to human communicative signals. Curr. Biol. 22, 209–212. (doi:10.1016/j.cub.2011.12.018) 22226744 10.1016/j.cub.2011.12.018

[37] Senju A, Csibra G, Johnson MH 2008 Understanding the referential nature of looking: infants' preference for object-directed gaze. Cognition 108, 303–319. (doi:10.1016/j.cognition.2008.02.009) 18371943 10.1016/j.cognition.2008.02.009

[38] Hare B 2001 Can competitive paradigms increase the validity of experiments on primate social cognition? Anim. Cogn. 4, 269–280. (doi:10.1007/s100710100084) 24777517 10.1007/s100710100084

[39] Hare B, Tomasello M 2004 Chimpanzees are more skillful in competitive than in cooperative cognitive tasks. Anim. Behav. 68, 571–581. (doi:10.1016/j.anbehav.2003.11.011)

[40] Bálint A, Faragó T, Meike Z, Lenkei R, Miklósi Á, Pongrácz P 2015 ‘Do not choose as I do!’—Dogs avoid the food that is indicated by another dog's gaze in a two-object choice task. App. Anim. Behav. Sci. 170, 44–53. (doi:10.1016/j.applanim.2015.06.005)

[41] Emery NJ 2000 The eyes have it: the neuroethology, function and evolution of social gaze. Neurosci. Behav. Rev. 24, 581–604. (doi:10.1016/S0149-7634(00)00025-7) 10.1016/s0149-7634(00)00025-7 10940436

[42] Phillips AT, Wellman HM, Spelke ES 2002 Infants' ability to connect gaze and emotional expression to intentional action. Cognition 85, 53–78. (doi:10.1016/S0010-0277(02)00073-2) 12086713 10.1016/s0010-0277(02)00073-2

[43] Tomasello M, Call J, Hare B 2003 Chimpanzees understand psychological states—the question is which ones and to what extent. Trends Cogn. Sci. 7, 153–156. (doi:10.1016/S1364-6613(03)00035-4) 12691762 10.1016/s1364-6613(03)00035-4

[44] Santos LR, Hauser MD 1999 How monkeys see the eyes: cotton-top tamarins’ reaction to changes in visual attention and action. Anim. Cogn. 2, 131–139. (doi:10.1007/s100710050033)

[45] Pongrácz P, Miklósi Á, Timár-Geng K, Csányi V 2004 Verbal attention getting as a key factor in social learning between dog and human. J. Comp. Psychol. 118, 375–383. (doi:10.1037/0735-7036.118.4.375) 15584774 10.1037/0735-7036.118.4.375

[46] Topál J, Gergely G, Erdöhegyi A, Csibra G, Miklósi Á 2009 Differential sensitivity to human communication in dogs, wolves, and human infants. Science 325, 1269–1272. (doi:10.1126/science.1176960) 19729660 10.1126/science.1176960

[47] Kis A, Hernádi A, Kanizsár O, Gácsi M, Topál J 2015 Oxytocin induces positive expectations about ambivalent stimuli (cognitive bias) in dogs. Horm. Behav. 69, 1–7. (doi:10.1016/j.yhbeh.2014.12.004) 25530486 10.1016/j.yhbeh.2014.12.004

[48] Elgier AM, Jakovcevic A, Barrera G, Mustaca AE, Bentosela M 2009 Communication between domestic dogs (*Canis familiaris*) and humans: dogs are good learners. Behav. Processes 81, 402–408. (doi:10.1016/j.beproc.2009.03.017) 19520240 10.1016/j.beproc.2009.03.017

[49] Udell MAR, Dorey NR, Wynne CDL 2008 Wolves outperformed dogs in following human social cues. Anim. Behav. 76, 1767–1773. (doi:10.1016/j.anbehav.2008.07.028)

[50] Wynne CDL, Udell MAR, Lord K 2008 Ontogeny's impacts on human–dog communication. Anim. Behav. 76, 402–408. (doi:10.1016/j.anbehav.2008.03.010)

[51] Bräuer J, Kaminski J, Riedel J, Call J, Tomasello M 2006 Making inferences about the location of hidden food: social dog, causal ape. J. Comp. Psychol. 120, 38–47. (doi:10.1037/0735-7036.120.1.38) 16551163 10.1037/0735-7036.120.1.38

[52] Gácsi M, McGreevy P, Kara E, Miklósi Á 2009 Effects of selection for cooperation and attention in dogs. Behav. Brain Funct. 5, 31–38. (doi:10.1186/1744-9081-5-31) 19630939 10.1186/1744-9081-5-31 PMC2731781

[53] Kaminski J 2009 Dogs (*Canis familiaris*) are adapted to receive human communication. In Neurobiology of ‘umwelt’: how living beings perceive the world (eds Berthoz A, Christensen Y), pp. 103–107. Berlin, Germany: Springer.

[54] Goossens BMA, Dekleva M, Reader SM, Sterck EHM, Bolhuis JJ 2008 Gaze following in monkeys is modulated by observed facial expressions. Anim. Behav. 75, 1673–1681. (doi:10.1016/j.anbehav.2007.10.020)

[55] Teufel C, Gutmann A, Pirow R, Fischer J. 2010 Facial expressions modulate the ontogenic trajectory of gaze-following among monkeys. Dev. Sci. 13, 913–922. (doi:10.1111/j.1467-7687.2010.00956.x) 20977562 10.1111/j.1467-7687.2010.00956.x

[56] Gergely G, Csibra G 2006 Sylvia's recipe: the role of imitation and pedagogy in the transmission of cultural knowledge. In Roots of human society: culture, cognition and human interaction (eds Enfield NJ, Levenson SC), pp. 229–255. Oxford, UK: Berg Publishers.

[57] Topál J, Miklósi Á, Sümego Z, Kis A. 2010 Response to comment on ‘Differential sensitivity to human communication in dogs, wolves, and human infants'. Science 329, 142-d (doi:10.1126/science.1184152) 10.1126/science.1184045 20616252

[58] Topál J, Kis A, Oláh K 2014 Dogs' sensitivity to human ostensive cues: a unique adaptation? In The social dog, behaviour and cognition (eds Kaminski J, Marshall-Pescini S), pp. 319–346. San Diego, CA: Academic Press.

[59] Tauzin T, Csík A, Kis A, Topál J. 2015 What or where? The meaning of referential human pointing for dogs (*Canis familiaris*). J. Comp. Psychol. 129, 334 (doi:10.1037/a0039462) 26147704 10.1037/a0039462

