# Supplementary material for: Do pet dogs (Canis familiaris) follow ostensive and non-ostensive human gaze to distant space and to objects?
Source: R Soc Open Sci. 2017 Jul 26;4(7):170349. doi: 10.1098/rsos.170349 (PMC5541559; doi:10.1098/rsos.170349)
Supplement: Table S1 and additional results [file rsos170349supp1.docx]

Table S1. Dogs who participated in the study

| **Dogs’ name** | Breed | Age | Sex | Exp1&2 | Exp3 |
| --- | --- | --- | --- | --- | --- |
| **Aikyo** | Leonberg | 5.5 | Female | yes | NO |
| **Aimy** | Border Collie | 6.5 | Female | yes | NO |
| **Amy** | Border Collie | 3 | Female | yes | O |
| **Ari** | Labrador | 5 | Male | yes | / |
| **Arwen** | Labrador | 10 | Female | yes | / |
| **Bacino** | Mixed | 2.5 | Male | yes | / |
| **Baris** | Border Collie | 8 | Male | yes | / |
| **Barney** | Mixed | 1.5 | Male | / | NO |
| **Benji** | Border Collie | 11.5 | Male | yes | O |
| **Benji** | Beauceron | 3 | Male | yes | NO |
| **Blake** | Border Collie | 1.5 | Male | yes | O |
| **Blue** | Berger hollandais | 2 | Female | yes | O |
| **Bonny** | Border Collie | 2.5 | Female | yes | O |
| **Boomer** | Border Collie | 3 | Female | yes | NO |
| **Cassy** | Daschund | 4 | Female | yes | O |
| **Caya** | Border Collie | 5.5 | Female | yes | / |
| **Charly** | Golden Retriever | 2.5 | Male | yes | O |
| **Charly** | Poodle | 8 | Male | yes | / |
| **Clea** | Shetland | 5 | Female | yes | NO |
| **Cliff** | Collie | 3.5 | Male | yes | / |
| **Connor** | Aussie | 4 | Male | yes | NO |
| **Cookie** | Border Collie | 1 | Female | yes | NO |
| **Curly** | Airedale Terrier | 10.5 | Female | yes | O |
| **Daisy** | Mixed | 2 | Female | yes | / |
| **Dean** | Border Collie | 2 | Male | yes | / |
| **Dublin** | German Shepherd | 9 | Female | yes | / |
| **Ellen** | Border Collie | 3 | Female | yes | / |
| **Emma** | Aussie | 3 | Female | yes | O |
| **Enja** | Dobermann | 10 | Female | yes | / |
| **Esmee** | Flat Coated Retriever | 3.5 | Female | yes | O |
| **Fenja** | Jack russel | 4.5 | Female | yes | / |
| **Fibi** | Mixed | 5 | Female | yes | NO |
| **Flappi** | Mixed | 3.5 | Female | yes | O |
| **Frozen** | Labrador | 5 | Female | yes | / |
| **Gustl** | French bulldog | 3.5 | Male | yes | / |
| **Heinrich** | Mixed | 7 | Male | yes | / |
| **Helena** | Great Poodle | 1.5 | Female | yes | NO |
| **Jane** | Jack Russel | 1.5 | Female | yes | O |
| **Jay** | Border Collie | 6.5 | Male | yes | O |
| **Joey** | Mixed | 6 | Male | yes | O |
| **Joy** | Labrador | 3 | Female | yes | / |
| **Juki** | Labrador | 7.5 | Male | yes | O |
| **Kimmy** | Mixed | 2.5 | Female | yes | O |
| **Kira** | Mixed | 5.5 | Female | yes | NO |
| **Kiwi** | Mixed | 2 | Female | yes | / |
| **Leila** | Mixed | 5.5 | Female | / | NO |
| **Lotti** | Fox terrier | 5 | Female | yes | / |
| **Maddy** | Mixed | 2 | Female | yes | / |
| **Mago** | Golden Retriever | 8 | Male | yes | / |
| **Marteja** | Mixed | 6.5 | Female | yes | / |
| **Milow** | Labrador | 2 | Male | yes | O |
| **Mopsi** | Pug | 5 | Female | yes | / |
| **Mozart** | Golden Retriever | 7.5 | Male | yes | O |
| **Nanouk** | Poodle | 5 | Male | yes | / |
| **Nele** | Bearded Collie | 7 | Female | yes | NO |
| **Nelly** | Labrador | 6 | Female | yes | / |
| **Nina** | Dalmatien | 8 | Female | yes | / |
| **Ophelia** | Braque Hongrois | 3 | Female | yes | NO |
| **Pooch** | Mixed | 5 | Female | yes | / |
| **Puk** | French bulldog | 6.5 | Male | yes | / |
| **Sam** | Border Collie | 4.5 | Male | yes | / |
| **Rocky** | Mixed | 7 | Male | / | NO |
| **Roger** | Mixed | 10.5 | Male | / | NO |
| **Sam** | Border Collie | 4.5 | Male | yes | NO |
| **Shadow** | Mixed | 9 | Female | yes | / |
| **Soferl** | Mixed | 5.5 | Female | yes | / |
| **Stasi** | Daschund | 8 | Female | yes | O |
| **Teddy** | Belgian sheperd | 6 | Male | yes | NO |
| **Theo** | German sheperd | 7.5 | Male | yes | NO |
| **Xena** | Jack russel | 9.5 | Female | yes | / |

In total 70 dogs were tested. 65 dogs were tested in Experiments 1 and 2, and 38 in Experiment 3. Some dogs were tested only in Experiments 1 and 2, some only in Experiment 3, depending on the owner’s availability. Order of Experiment 1 and 2 was counterbalanced across dogs. When a dog was tested in all three experiments, Experiment 3 was always conducted after Experiments 1 and 2. In experiment 3: NO= Repetitive Non-Ostensive condition; O= Repetitive Ostensive condition.
